# Establishment from seed is more important for exotic than for native plant species

**DOI:** 10.1002/pei3.10132

**Published:** 2023-12-07

**Authors:** Brian Wilsey, Andrew Kaul, H. Wayne Polley

**Affiliations:** ^1^ Department of Ecology, Evolution and Organismal Biology Iowa State University Ames Iowa USA; ^2^ Center for Conservation and Sustainable Development Missouri Botanical Garden St. Louis Missouri USA; ^3^ Grassland, Soil and Water Research Laboratory USDA‐ARS Temple Texas USA

**Keywords:** alternate states, Blackland Prairie, community assembly, ecosystem conversion, invasive species, non‐native species, *Panicum coloratum*, seed limitation, *Sorghum halepense*, tallgrass prairie

## Abstract

Climate change has initiated movement of both native and non‐native (exotic) species across the landscape. Exotic species are hypothesized to establish from seed more readily than comparable native species. We tested the hypothesis that seed limitation is more important for exotic species than native grassland species. We compared seed limitation and invasion resistance over three growing seasons between 18 native and 18 exotic species, grown in both monocultures and mixtures in a field experiment. Half of the plots received a seed mix of the contrasting treatment (i.e., exotic species were seeded into native plots, and native species were seeded into exotic plots), and half served as controls. We found that (1) establishment in this perennial grassland is seed limited, (2) establishment from seed is greater in exotic than native species, and (3) community resistance to seedling establishment was positively related to diversity of extant species, but only in native communities. Native‐exotic species diversity and composition differences did not converge over time. Our results imply that native to exotic transformations occur when diversity declines in native vegetation and exotic seeds arrive from adjacent sites, suggesting that managing for high diversity will reduce transformations to exotic dominance.

## Graphical abstract

Seed dispersal and seedling establishment is higher in non‐native plant species than it is in native species. Seedling establishment is higher in native monocultures than it is in diverse mixtures.

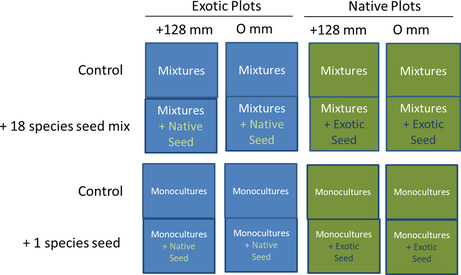

## 1 INTRODUCTION

Climate change will initiate movement of both native and non‐native (exotic) species across the landscape, and establishment from seed is expected to become increasingly important (Iseli et al., 2023; McConkey et al., 2012). Dispersal among patches is considered to be one of the major forces behind community assembly, linking local communities into metacommunities (Leibold et al., 2004; Vellend, 2010). In prairies, plants commonly disperse into new patches via vegetative ramets (Benson & Hartnett, 2006), but seeds are especially important for longer‐distance dispersal events (Foster et al., 2004; Myers & Harms, 2009). Seedling establishment during these events will become increasingly important as species shift in response to climate change. Seed dispersal distances vary greatly across grassland species (Sullivan et al., 2018), ranging from <1 m in non‐plumed species to >1 m in plumed species. Seeds can also travel on the fur of animals, sometimes traveling great distances in the process (Balzinger et al., 2019; Eyheralde, 2015). Seeds can have difficulty arriving in habitat patches in human‐dominated environments, where habitat exists as small islands far apart from one another in a larger matrix (Holyoak et al., 2020). Seeds may also have different establishment success depending on whether patches are dominated by native or non‐native (exotic) species, and whether the dispersing species are native or non‐native (Bucharova & Krahulec, 2020; Suding & Gross, 2006; Togneti & Chaneton, 2012; Wainwright & Cleland, 2013).

Exotic species may differ from native species in their establishment from seed (Charles et al., 2022; Gioria & Pyšek, 2017; Martin et al., 2014; Seabloom et al., 2015; Wilsey et al., 2011). Exotic species often have higher germination rates than phylogenetically related native species and are often active earlier in the growing season (van Kleunen et al., 2010; Wainwright & Cleland, 2013; Wilsey et al., 2015). Wilsey et al. (2015) compared 14 native and 14 exotic plant grassland species from the central USA, with species being paired by phylogeny and functional group. They found that exotic species had approximately two times higher seed germination rates and quicker emergence times, with the first seedling emerging in half the number of days compared to native species. Traits related to early growth could enable exotics to more readily invade native‐dominated areas (Charles et al., 2022; Chrobock et al., 2011). After invasion, exotics could lower resources (Daneshgar et al., 2013; Wainwright & Cleland, 2013; Wilsey et al., 2023), impede establishment of native seedlings, and eventually form a “stable” exotic state that persists over time. However, if exotics are associated with reduced species diversity, this, in turn, could lead to higher invasion rates.

Greater recruitment from arriving seeds in low‐diversity patches could lead to a convergence in diversity and composition between high‐diversity native patches and low‐diversity exotic patches, although this has seldom been tested. In many perennial grassland systems, native‐dominated areas have higher diversity than non‐native‐dominated areas (Hejda et al., 2009); diversity could potentially alter the likelihood of seedling establishment independently of native‐exotic status. High species diversity at the plant neighborhood scale has often been found to improve invasion resistance in experiments that have varied biodiversity (e.g., Hooper et al., 2005; Kennedy et al., 2002; Weisser et al., 2017; Wilsey & Polley, 2002; Zavaleta & Hulvey, 2004). Reduced species diversity could lead to unused resources that are then available to invaders (Foxcroft et al., 2011). The few tests of convergence from seed additions so far have found that differences do not converge (Kulmatiski, 2006; Martin & Wilsey, 2014; Schröder et al., 2005).

The result of species interactions and altered seedling recruitment could be alternate states of low‐diversity exotic vegetation intermixed with diverse patches of native species (Kulmatiski, 2006; Stotz et al., 2020). These patches differ in two ways, their native‐exotic compositional difference and their levels of species diversity, both of which could potentially affect seedling establishment and should be measured in experiments. Lower diversity exotic‐dominated stands certainly exist adjacent to diverse, native‐dominated stands (e.g., Martin & Wilsey, 2014; Stotz et al., 2020; Wilsey et al., 2023), but it is not always known if these states are caused by the exotic species themselves or by associated disturbances (Hejda et al., 2009; MacDougall & Turkington, 2005). That is, it is not known whether state differences are causal in nature. Seed additions of native species into exotic patches failed to increase seedling recruitment and native species abundance in grassland restoration plantings (Martin & Wilsey, 2014), but seeds of exotic species have rarely been added to native patches due to ethical concerns. Without seed addition experiments, we do not know if native stands have high‐diversity and low‐exotic abundance because seeds of exotic species have not arrived at the site or due to other associated variables. Conversely, we also do not know whether exotic stands have low‐diversity and low‐native species abundance because of seed limitation by native species. Alternate states are only considered stable if they have resisted establishment from members of the other state (Fukami, 2015; Schröder et al., 2005).

We compared seed limitation and invasion resistance between 18 native and 18 exotic plant species growing in both monocultures and mixtures in a long‐term central Texas field experiment. We conducted seed additions to test whether these states were stable (resistant to invasion). Half of the plots received a seed mix of the contrasting treatment (i.e., exotic species were seeded into native plots, and native species were seeded into exotic plots), and half served as controls. Our hypotheses were that: (1) seed limitation exists for both native and exotic species, (2) seed limitation is more important in exotic species than comparable native species, and (3) the high‐diversity native state and low‐diversity exotic state are stable (i.e., resistant to invasion and will not converge in species composition and diversity with seed additions). Number of seedlings of native and exotic species, species composition, and species richness were measured over time to test these hypotheses.

## 2 METHODS

### 2.1 Experimental design

The study involved adding seeds to plots in an ongoing long‐term experiment that compared native and exotic plant communities under ambient and enhanced precipitation. The MEND experiment (Maintenance of Exotic vs. Native Diversity) was established in 2008 near Temple, Texas to test how species diversity and ecosystem processes differed between grassland plots dominated by all exotic vs. all native plant species, with native vs. exotic treatments crossed with summer irrigation treatments (Wilsey et al., 2011). In total, 64 mixture plots and 144 monoculture plots, each 1 m × 1 m, were established. Half of the plots were irrigated yearly by adding 128 or 0 mm of tap water during the normally driest period of the year (July 15 to August 15). Irrigation was added twice per week in 16 mm increments. Native versus Exotic treatments were applied to plots by populating the plots with 72 equal‐sized transplants of either 9 native or 9 exotic plant species using 8 random draws of species from a pool of 18 native or 18 exotic plant species. Random draws were made to include all four functional groups (C_4_ grasses, C_3_ grasses, Forbs, Leguminous forbs) in each plot, with native and exotic species paired by functional group and phylogeny (Wilsey et al., 2011). By the second year of the study, these treatments had developed into a high diversity state in native plots, and a low diversity state in exotic plots (Wilsey et al., 2011). Thus, there were 2 (native or exotic) × 2 (irrigated or not) × 2 blocks × 4 draws per block × 2 replicates = 64 mixtures, and 18 species × 2 (native or exotic) × 2 (irrigated or not) × 2 blocks = 144 monocultures. For further details on the experiment, see Wilsey et al. (2011, 2014) and Xu et al. (2017).

**FIGURE 1:**
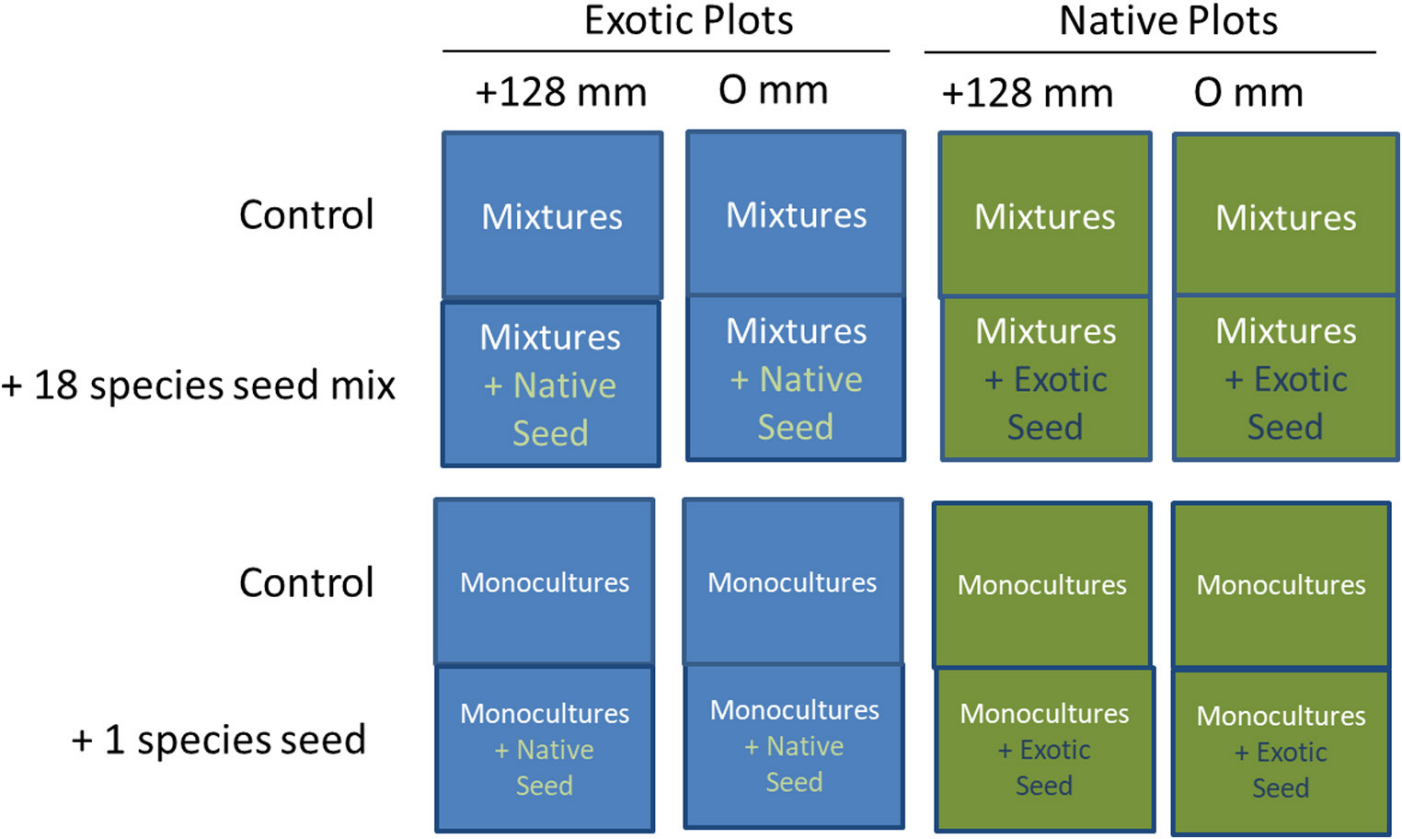
Experimental design for the seed addition experiment. Seed addition treatments were added to an ongoing experiment with 208 plots that compared all native species plots to all exotic species plots, with and without summer irrigation (0, +128 mm), using phylogenetically‐paired native and exotic species. Half of the 64 mixtures received an 18 species seed mix of the corresponding pair (natives into exotic plots, exotics into native plots). Half of the 144 monocultures received a 1‐species addition of the phylogenetically paired native or exotic species (i.e., native into exotic and exotic into native).

Here, we report on a seed addition experiment to existing plots (Figure 1). Exotic plots were dominated by *Sorghum halepense*, *Panicum coloratum*, *Bothriochloa ischaemum*, or *Cynodon dactylon*, and native plots were a higher diversity mixture of grasses and forbs at the onset of the seed addition experiment. Seeds were added in October 2016. Seedling recruitment is more common in the winter and early spring in our area compared to other seasons (Wilsey & Polley, 2003). None of the plots had been harvested when the seeds were added. Plots were randomly segregated into two groups, and one of the groups (half the mixtures, *n* = 32, and half the monocultures, *n* = 72) received seed additions and one of the groups (*n* = 32 mixtures and *n* = 72 monocultures) did not. Seeds were added at an amount of 8110 seeds per 1 m^2^ plot (450 per species), using a value that was higher than some other seed addition studies (e.g., Dickson & Foster, 2008 added 300 seeds per species). Seed was pure live seed purchased from Native American Seed Co. (all native species, Junction, TX) and a variety of other seed companies for exotic species. Seed was purchased rather than using hand‐collected seed from the field for two reasons: (1) to keep all species comparable as some were not locally available in field plots, and (2) to make results comparable to past and current seedings that use purchased seed (note that most of these species were added as seeds from seed companies in the past and present). High seed rates were used to mimic a typical seed rain in exotic‐dominated old fields (~7200–9200 per m^2^, Schott & Hamburg, 1997) and prairies (e.g., >19,000 seeds per m^2^, Rabinowitz & Rapp, 1980). Native mixtures received 8110 seeds of a seed mixture of all 18 exotic species combined. Exotic mixtures received 8110 seeds of a mix of all 18 native species combined. Each seeded monoculture was seeded with the phylogenetically‐paired native or exotic species of each pair (Table S1) at a rate of 8110 seeds per m^2^. For example, plots of the exotic Johnson grass (*Sorghum halepense*) either were seeded with the native Indian grass (*Sorghastrum nutans*) or not seeded and Indian grass plots received either Johnson grass seed or no seed. This paired addition treatment was repeated for each of the 17 other species pairs (Table S1).

### 2.2 Sampling design and statistical analyses

Seedlings were counted by species during June (peak of the C_3_ production period) and October (peak of C_4_ production period) over three growing seasons following seed additions (five sampling dates total). Species richness and presence of the originally planted species, as well as the number of not‐seeded (volunteer invader) individuals, were also measured at each sampling. Data on seedling counts were analyzed with a generalized linear model with a log‐link function and a Poisson distribution using Proc GLYMIXX in SAS 9.4 (Bolker et al., 2009).

We also determined how species diversity within mixture type (native or exotic) affected seedling establishment by comparing mixtures (originally planted with nine species) and their corresponding monocultures (one species). This was tested by comparing the number of seeded plants in mixtures (observed; O) to the expected number of seeded plants in monocultures (expected; E). We compared seedling numbers in mixtures and monocultures with a Net Biodiversity Index (NBI) for each mixture *i*, calculated as the number of seedlings in each *i* mixture (*O*)—expected, where expected is the number of seedlings in monocultures (*Es*) of each species *s* present:

$$\mathrm{NBI}i=\mathrm{Oi}-\sum_{s=1}^{18}\mathrm{Es}\times 1/18.$$

This measure compares species at low (monocultures) and high diversity (mixtures) to test the hypothesis that establishment will be lower in low‐diversity plots. Eighteen species were included in the seed mixes, so E was divided by 18. This calculation produced 64 values, one for each mixture. Negative values of NBI indicate that seedling emergence was lower in a given mixture than in monocultures of the species present in the mixture. NBI was analyzed with mixed model repeated measures ANOVA after ln transformation (but with retaining signs, Loreau and Hector (2001), with seed additions, irrigation, and origin as fixed effects). Interactions across time periods were analyzed with mixed model repeated measures ANOVA with seeded plots only to test how seed addition effects changed with time.

We used the trajectory analysis approach of De Caceres et al. (2019) and permutational MANOVA to test the hypothesis that community types (native vs. exotic) would converge over time with the seed addition treatments (seeds added or not, four treatments total). Convergence is found with a decrease in distance over time and eventually, a non‐significant PERMANOVA term. To test for convergence, we used the frequency of species' occurrence among plots within treatments, calculated Bray–Curtis distances between treatments on each sampling date, and conducted a Mann‐Kendall trend test with the trajectoryconvergence() function in the “ecotraj” R package (De Caceres et al., 2019). We used three‐way PERMANOVA based on Bray–Curtis distances to test whether community composition in the mixture plots was different among the two addition (Added, Control), two origin (Native, Exotic), and two irrigation (Irrigated, Control) treatments (fixed effects) for each sampling date using Primer 7 software (Anderson, 2017). Rep(Add × Orig × Irr) was the random effect error term for the main effect treatments, and the residual was used for date and interactions with date. The irrigation term and the interaction between treatment and irrigation were non‐significant on all sampling dates (see Section 3), so terms were pooled into the error term and the final analysis and ordinations were performed on main effect and two‐way interactions alone. We visualized treatment‐level trajectories in community composition based on presence/absence data using Principal Coordinates Analysis (Vegan package of R).

## 3 RESULTS

**FIGURE 2:**
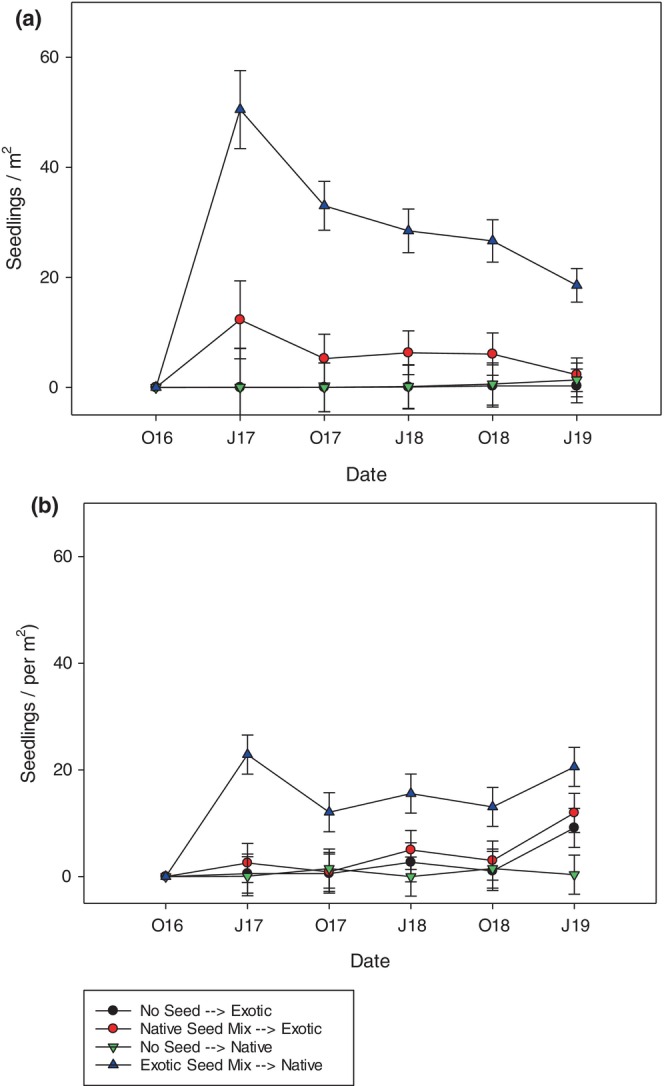
Mean number of seedlings of seeded species in plots in June (J) and October (O) of 3 years in monoculture plots (a) and mixture plots (b). In “(a),” single native species were seeded into paired exotic monocultures (Native—Seeds, red bar), and single exotic species were seeded into paired native monocultures (Exotic—Seeds, blue bar), or plots were left unseeded (No seed Controls, black and green bars, denoted “None”). In “(b),” exotic seed mixes were added into native species mixture plots, native seed mixes were added into exotic species mixtures plots, or mixtures were left unseeded (“No Seed” Controls).

Overall, exotic seedling counts were consistently much higher than native species seeded into both established mixtures and monocultures (Figure 2). Addition of seeds led to 2× to 4× greater seedling numbers for exotic species seeded into native plots than native species seeded into exotic plots (*p* < .001). This result was consistent across the five sampling dates (Figure 2), and resulted in exotic plots being more resistant to invasion than native plots.

### 3.1 Single species seed additions into monocultures

Addition of seeds to established monocultures led to much higher seedling emergence compared to controls in both exotic and native plots, indicating significant seed limitation (Figure 2a; Figure S1). Exotic species established into native species monocultures much more abundantly than did native species into exotic monocultures. The number of exotic seedlings that emerged in native monocultures was roughly fivefold greater than the number of native seedlings that emerged in exotic monocultures. By the third growing season, all added species had reached maturity and were flowering (personal observation).

Addition of seeds of single exotic species heavily invaded native monocultures in 9 out of 18 cases (i.e., there were many more exotic seedlings than its comparable native species and percent cover was high for these species [Figure S1 and personal observation]). Phylogenetically‐paired native species invaded exotic monocultures in only 2 of 18 cases (Figure S1). Exotic species that were successfully established in native monocultures were from all functional groups but were dominated by C_3_ species (grasses, forbs, and legumes). Many of these species had been outcompeted previously from exotic mixtures (Wilsey et al., 2011).

### 3.2 Seed additions into mixtures

Seed additions also significantly increased seedling numbers in mixtures (Figure 2b; Table 1), but fewer plants of seeded species were evident in mixtures than in monocultures (Figure 2a). Again, exotic seedlings were established in greater numbers in native plots than did native seedlings into exotic plots (Figure 2b). The effect of seed additions and the native‐exotic differences were consistent across the five samples. The native grass species *Nasella leucotricha* successfully invaded exotic plots regardless of seed addition treatments, resulting in many native seedlings in exotic plots on the last date (Figure 2b).

**TABLE 1:** Fixed effects results for number of seedlings in a generalized linear model with a Poisson distribution.

	df	*F*	*p*
Mixtures			
Origin	1, 56	0.4	.52
Irrig	1, 56	0.2	.65
Origin × Irrig	1, 56	0.3	.62
**Seed Added**	**1, 56**	**61.6**	**<.0001**
**Origin × Added**	**1, 56**	**20.3**	**<.0001**
Irrig × Added	1, 56	0.5	.51
Origin × Irrig × Added	1, 56	0.04	.85
**Date**	**4, 244**	**60.2**	**<.0001**
**Added × Date**	**4, 244**	**10.4**	**<.0001**
**Origin × Date**			
Fit: *λ* ^2^ = 2.6	**4, 244**	**43.8**	**<.0001**
Monocultures			
**Origin**	**1, 117**	**3.8**	**.05**
Irrig	1, 117	0.1	.80
Origin × Irrig	1, 117	0.02	.90
**Seed Added**	**1, 117**	**49.2**	**<.0001**
Origin × Added	1, 117	2.9	.09
Irrig × Added	1, 117	0.6	.45
Origin × Irrig × Added	1, 117	0.1	.72
**Date**	**5,565**	**7.5**	**<.0001**
**Added × Date**	**5, 565**	**10.9**	**<.0001**
**Origin × Date**	**5, 565**	**27.6**	**<.0001**
Fit: *λ* ^2^ = 3.5			

*Note*: Origin denotes difference between native and exotic plots, irrig. denotes plots that were irrigated with 128 mm of water per year versus 0 mm of water per year, Added denotes plots that received seed additions or did not, and Date is five dates over 3 years. Significant terms are bolded (*p* < 0.05).

**FIGURE 3:**
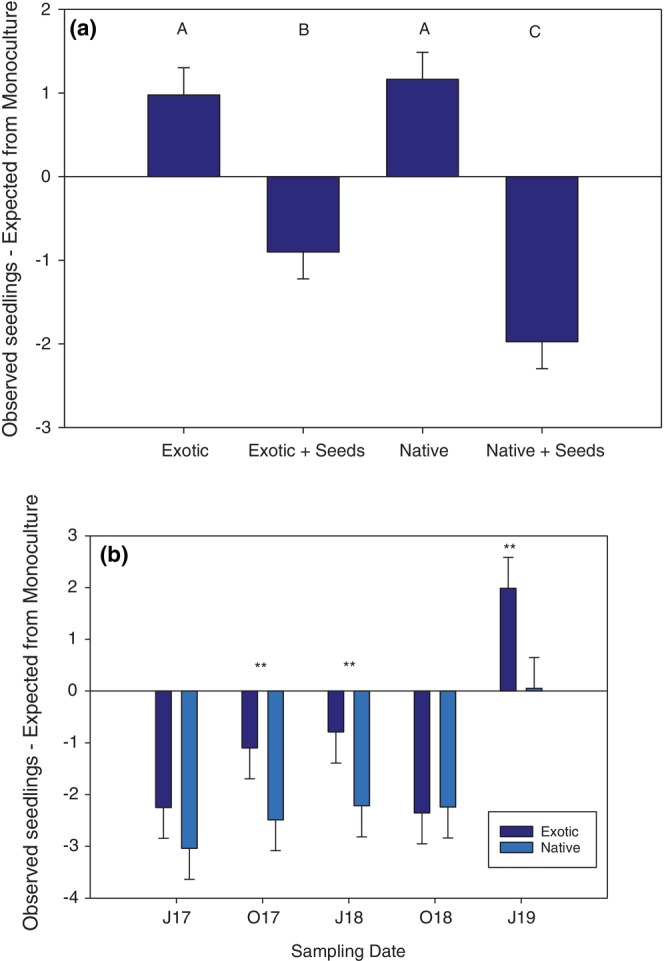
(a) Mean difference in number of seedlings in mixtures compared to expectations from monocultures (NBI) for unseeded exotic plots (Exotic), exotic plots seeded with a native‐species seed mix (Exotic + Seeds), unseeded native plots (Native), and native plots seeded with an exotic‐species seed mix (Native + Seeds). Negative values denote a reduction in number of seedlings in mixtures compared to monocultures, and values are means + SE. (b) Mean NBI across sampling dates (+SE) for Exotic and Native mixtures that were seeded only. Asterisks denote *p* < .05 in tests by date.

Establishment from exotic species in native mixtures was reduced by higher species diversity. NBI was significantly more negative in native than exotic mixtures when seeded (Figure 3a). Exotic mixtures exhibited a relatively small reduction in seedlings compared to expected values from monocultures (Figure 3a). Native mixtures had a much larger reduction in the number of seedlings compared to expectations from monocultures through the first two growing seasons (Figure 3b). On the final date, NBI was significantly positive in exotic plots when *Nasella leucotricha* invaded.

**FIGURE 4:**
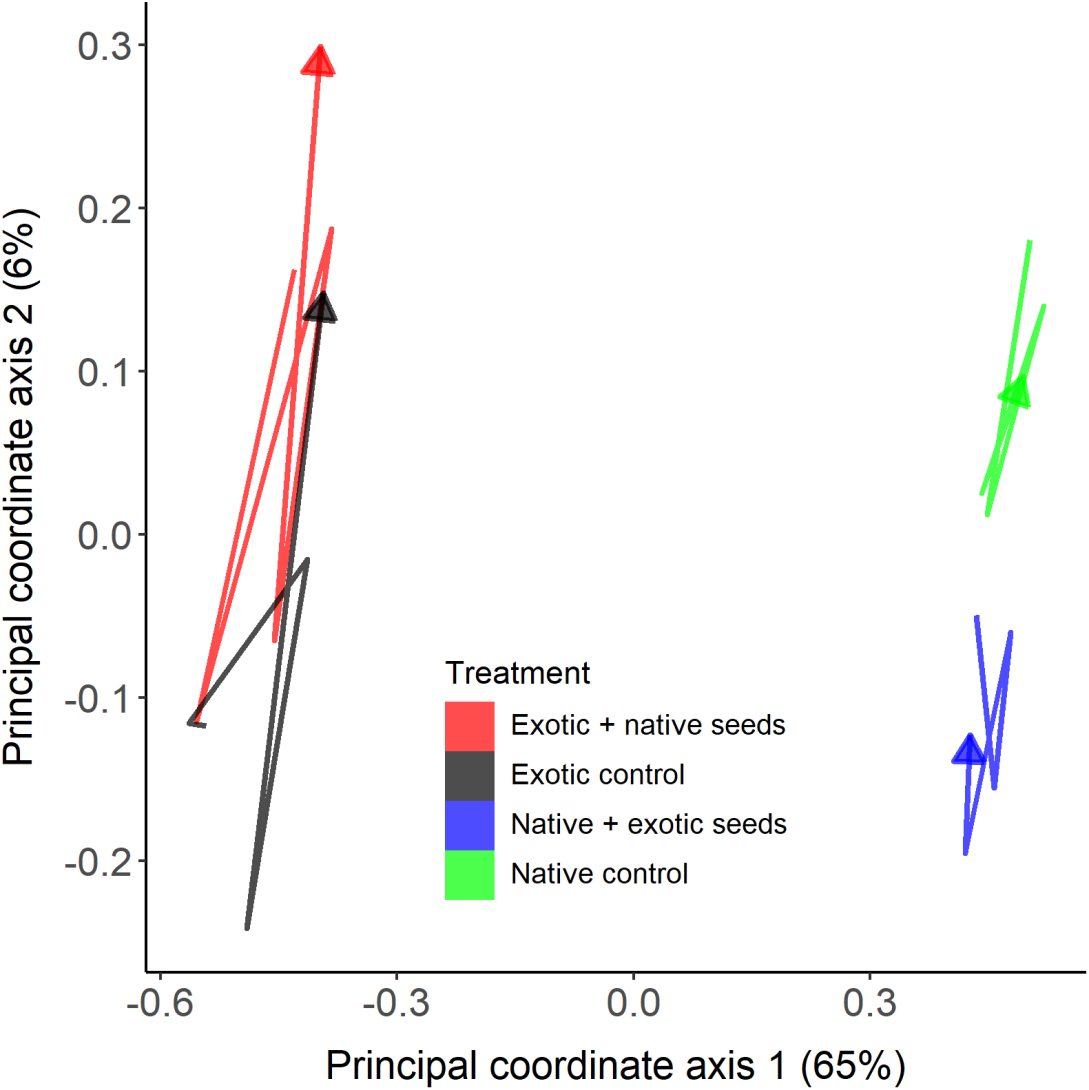
Trajectory analysis in species composition using Principal Coordinates Analysis (PCoA) over five time periods after seed additions in exotic plots that received a native species seed mix (Exotic + native seeds) or not (Exotic control), and in native plots that received an exotic seed mix (Native + exotic seeds) or not (Native control).

**FIGURE 5:**
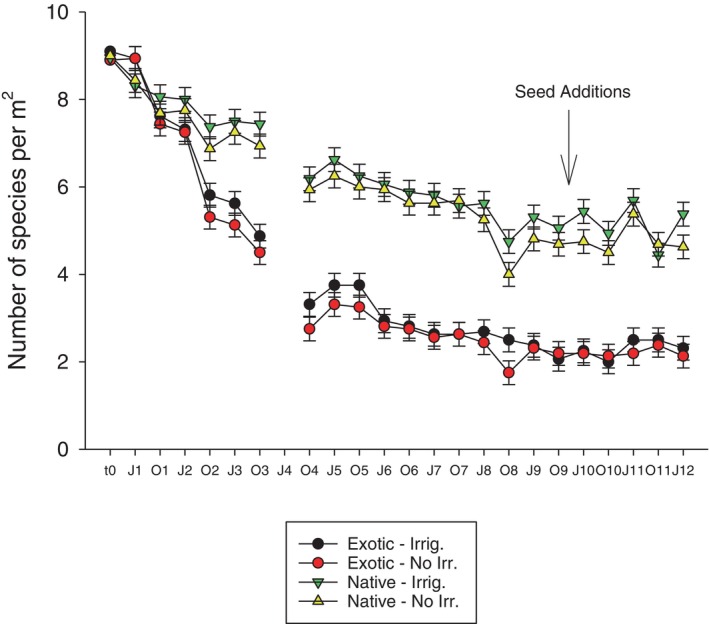
Species richness of planted species over 12 years in species mixtures of all exotic species or all native species, either irrigated with 128 mm per year (Irrig.) or non‐irrigated (No Irr.). Arrow denotes when seeds were added in the seed addition experiment.

Seed additions did not lead to convergence in species composition nor richness over time (Table 2; Figures 4 and 5). Treatments did not cause divergence over time in composition of exotic (*τ* = 0.2, *p* = .806), or native origin (*τ* = 0.4, *p* = .462) plots compared to their controls, nor did they cause convergence between native and exotic plots that received reciprocal species additions (*τ* = −0.2, *p* = .806). A PCoA ordination found that community trajectories did not move toward each other over time (Figure 4). The PERMANOVA analysis found only differences in composition between native and exotic mixtures, and across dates, and the native‐exotic differences persisted throughout the study (i.e., there were no significant interactions with date). This resulted in trajectory lines that moved parallel to exotic plots in ordinations (Figure 4).

**TABLE 2:** PERMANOVA results that compared exotic plots that had native seeds added (exotic addition), or not (exotic control), and native plots that had exotic seeds added (native addition), or not (native control).

Terms	df	Pseudo *F*	*p*
Added seeds	*1*	*1.3*	*.249*
**Native vs. Exotic (N/E)**	1	14.6	**.001**
Irrigation (Irr.)	1	0.4	.928
Added × N/E	1	1.4	.191
Added × Irr.	1	0.3	.974
Native vs. Exotic × Irr	1	0.6	.702
Rep(Added × N/E × Irr) random error term	56		
**Date**	4	344.3	**.001**
Addition × Date	4	0.3	.929

*Note*: Seed additions were made in Fall 2016. Significant terms are bolded (*p* < 0.05).

**FIGURE 6:**
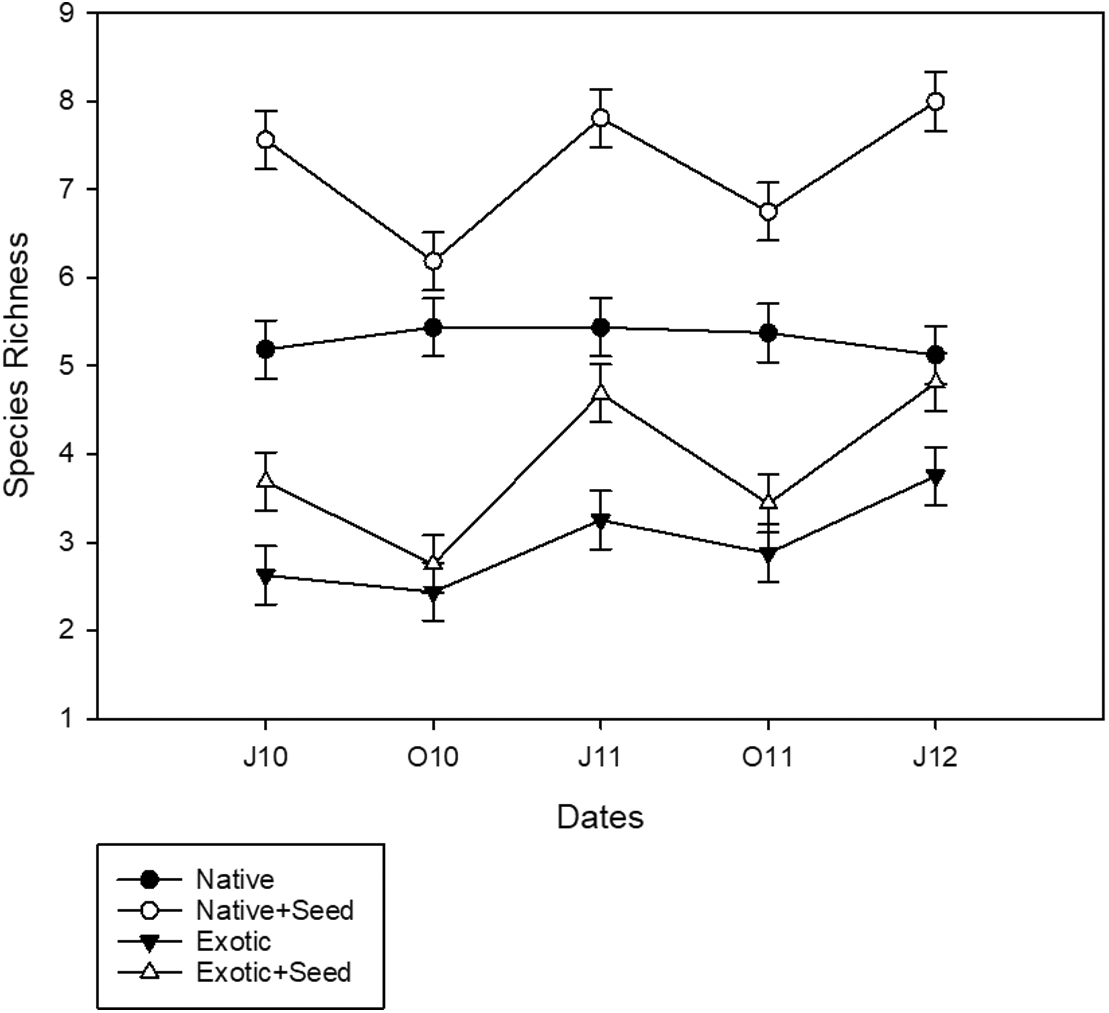
Plant species richness (mean ± SE) over three growing seasons in Native mixtures, Native mixtures with an exotic seed mix added (Native + Seed), Exotic mixtures, and Exotic mixtures with a native seed mix added (Exotic + Seed).

The difference between native and exotic species richness of the originally planted species persisted through to the end of the experiment (Figure 5). The seed additions did, however, result in an increase in new species from seed additions. The number of seeded species was greater for exotic species in native mixtures than it was for native species in exotic mixtures (Figure 6). Native mixtures had, on average, 0.2 new exotic species emerging when not seeded, and 2.4 new species of exotics when seeded. Exotic controls had, on average, 0.9 new native species emerging when not seeded, and 1.5 new species when seeded. These newly recruited species affected both total species richness and native‐exotic proportions. In contrast to the hypothesis that native‐exotic differences would converge due to seed additions, the differences in richness between native and exotic mixtures actually grew larger. By the end of the study, the native‐exotic richness difference between seeded mixtures (8 vs. 4.8) was larger than it was between unseeded mixtures (5.1 vs. 3.8) (Figure 6).

## 4 DISCUSSION

We found evidence that establishment in this perennial grassland is seed limited, and that establishment from seed is greater in exotic than native species. Exotic species more readily established from seed in extant mixtures of native species than the converse, consistent with our hypothesis. This suggests that exotic species may be spreading more from seed than native species in grassland landscapes. This has important implications to how plants will respond to climate change as seedling establishment in distant locations becomes more important. Exotic species will spread northward more easily than comparable native species, and native species will have difficulty establishing in exotic patches without disturbance. Community resistance to seedling establishment was higher in mixtures than it was in monocultures, and diversity mattered more in reducing seedling establishment for native than exotic grassland vegetation. On the other hand, diversity had less of an effect in exotic plots, as low‐diversity exotic mixtures contained fewer native seedlings than did high‐diversity native mixtures, contrary to the hypothesis that community resistance to establishment would be positively related to diversity. This indicates that native‐exotic differences in seedling establishment over‐rode the effects of diversity per se. Diversity was only important in reducing seedling establishment in native mixtures. Reduced species diversity in native prairie relicts is likely to leave these sites more vulnerable to invasion and conversion from native to exotic dominance. Somewhat surprisingly, summer irrigation treatments had very small effects on seedling establishment in both native and exotic species.

The exotic species that were most successfully established from seed in native mixtures were not the grasses that dominated exotic mixtures. Exotic mixtures were all dominated by C_4_ grasses (Martin et al., 2014; Wilsey et al., 2014; Xu et al., 2017). Yet, the most successful exotic species established in native mixtures from seed were forbs and C_3_ grasses. These and previous results when taken together, indicate that the exotic C_4_ grasses (Klein grass *Panicum coloratum*, Johnson grass *Sorghum halepense*, KR bluestem *Bothriochloa ischaemum*, and Bermuda *Cynodon dactylon*) more negatively impact abundances of exotic forb species than do comparable native C_4_ grasses.

High‐diversity native prairie sites are often surrounded by low‐diversity exotic sites in our current landscapes (Stotz et al., 2020; Wilsey et al., 2011). These two states differ both in their diversity (high, low) and in their composition (native vs. exotic species). Here, we tested whether the high‐diversity native vs. low‐diversity exotic states would collapse once seeds arrived from native species into exotic plots and vice versa. Our results indicate that the “stability” of the states depend on whether we consider species richness or composition. The initial differences in species richness persisted throughout the seed additions, indicating that the native and exotic mixtures represent stable states. Differences in species richness between native and exotic mixtures grew *larger* due to the seed additions, opposite of what we would expect if richness levels were converging. Longer time frames might provide a different result, but at least in the medium‐term length of our study (Clark et al., 2007), these different states persisted. Furthermore, species compositions between native and exotic plots did not converge over time after seed additions. There were some exotic species that successfully established into native plots, changing their native‐exotic proportions. This indicates that the 100% native state is not resistant to exotic species invasion and that over longer time frames, these exotic species could eventually affect native species composition and diversity.

The low establishment of native species into exotic patches has important implications for restoration. Previous restoration projects that over‐seeded exotic‐dominated patches to restore high native species diversity often found higher species richness and native species abundance, especially with disturbance (Orrock et al., 2009; Suding & Gross, 2006; Togneti & Chaneton, 2012; Zamin et al., 2017). Bucharova and Krahulec (2020) found that seeding native species led to high native grass establishment that in turn, reduced the spread of an exotic *Rumex* sp. A previous study near our field site found essentially zero establishment in fields dominated by *Bothriochloa ishaemum* (Wilsey et al., 2011). Here, we found little native species seedling establishment in plots with this C_4_ grass species, and plots dominated by *Sorghum halepense* and *Panicum coloratum*. Our results suggest that native seeding in this system will have low success without a disturbance treatment such as litter removal or mowing (Zamin et al., 2017). Further research is needed on how disturbances might interact with native seed additions in restorations (Wilsey & Martin, 2015).

**FIGURE 7:**
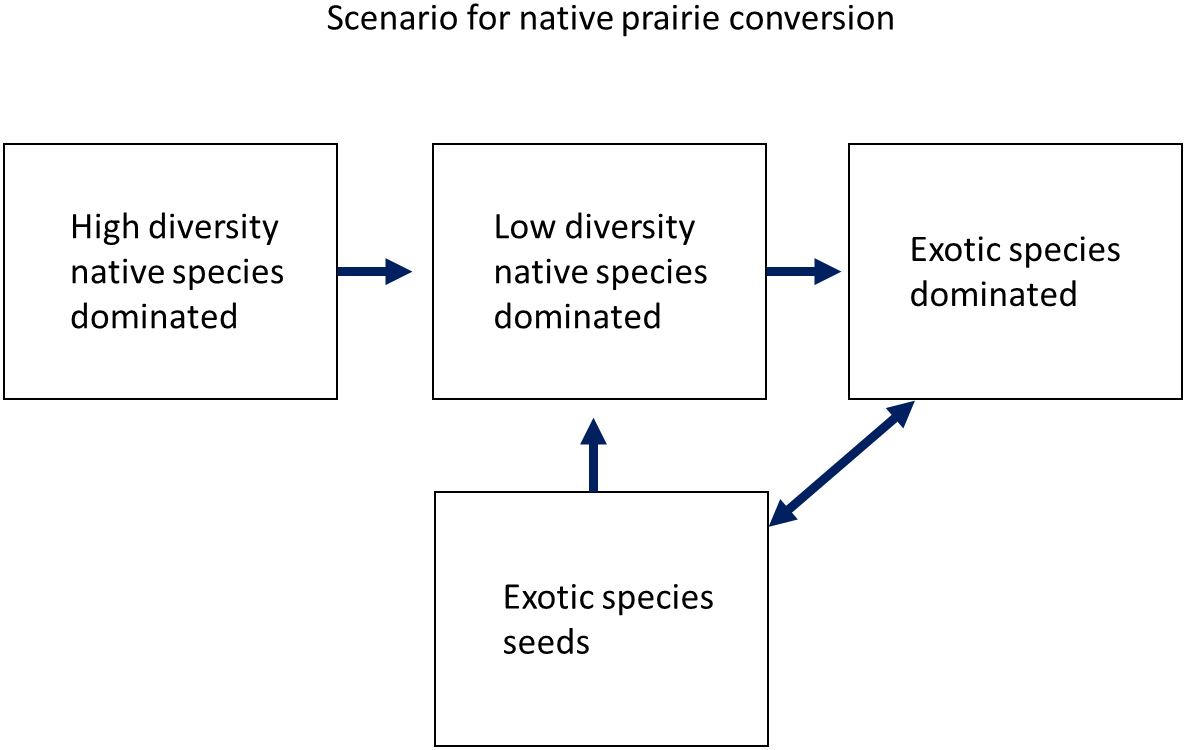
Scenario for change from native to exotic species dominance based on the results of this study. Native plots that had low species diversity (monocultures and low diversity mixtures) had high rates of establishment by exotic species, which suggests that reductions in diversity in native sites precedes conversion. Once exotic species invade from seed, a seed bank would maintain exotic species dominance.

Based on our results, we propose a mechanistic scenario for native prairie conversion to exotic species dominance (Figure 7). If native patches have high diversity, as our native mixtures do, the patch will have relatively low invasion by nearby exotic species. However, if diversity is reduced for some reason, the patch becomes more vulnerable to invasion by exotics (Maron & Marler, 2008; Xu et al., 2015). Heavy grazing, nutrient enrichment (Harpole et al., 2016), human planting of single native species, and broad‐leafed herbicides (Fuhlendorf et al., 2002) can all lead to reduced diversity. Reduced native diversity, in turn, could lead to exotic establishment into native sites, and eventually, a switch from native to exotic species dominance. Once exotic species establish and develop an exotic seed bank (Gioria & Pyšek, 2016, 2017; Yurkonis et al., 2005), the exotic species state will be maintained due to their higher seed germination rates (van Kleunen et al., 2010; Wilsey et al., 2015, this study). Projected changes to climate in this region may exacerbate this positive feedback, as non‐native species can be less affected by heating than native species, especially in disturbed soils (Haeuser et al., 2017).

In conclusion, our results indicate: (1) that seed dispersal is a stronger force among exotic than native species in grassland communities, and (2) that low diversity in native communities will increase their probability of being converted to exotic species dominance. Combining low species diversity with increased propagule pressure from exotic species could result in conversion of native‐dominated sites to exotic dominance. This new exotic state would then be reinforced, at least in disturbed environments, by the increased recruitment of exotic species from seed compared to natives (Gioria & Pysek, 2017; van Kleunen et al., 2010; Wainwright & Cleland, 2013; Wilsey et al., 2015). How this translates to measurements of plant movements in response to climate change should be studied in the future (Iseli et al., 2023).

## CONFLICT OF INTEREST STATEMENT

The authors have no conflict of interest.

## Supporting information


Data S1.

## Data Availability

Data is available in Dryad under https://doi.org/10.5061/dryad.k3j9kd5fh.
