# Supplementary material for: Establishment from seed is more important for exotic than for native plant species
Source: Plant Environ Interact. 2023 Dec 7;5(1):e10132. doi: 10.1002/pei3.10132 (PMC10840371; doi:10.1002/pei3.10132)
Supplement: Supplementary file 1 — Data S1. [file PEI3-5-e10132-s001.docx]

Supplemental Figure 1. Average number of seedlings per plot across native-exotic species pairs. Species pairs are numbered as follows: C_4_ grasses - 1. *Schizachyrium scoparium - Bothriochloa ischaemum, 2. Buchloe dactyloides - Cynodon dactylon, 3. Sporobolus asper - Eragrostis curvula, 4. Panicum virgatum - Panicum coloratum. 5. Eriochloa sericea - Paspalum dilatatum, 6. Sorghastrum nutans - Sorghum halapense;* C_3_ grasses - 7. *Nasella luecotricha - Dactylus glomerate 8. Elymus canadensis - Festuca arundinacea;* C_3_ *forbs 9. Ratibida columnifera - Leucanthemum vulgare, 10. Marshallia caespitosa -Taraxacum officinale 11. Vernonia baldwinii - Cichorium intybus, 12. Salvia azurea - Nepata cataria 13. Ruellia humilis - Ruellia brittoniana, 14. Monarda fistulosa - Marrubium vulgare,* C_3_ *Leguminous forbs:*

*15. Dalea purpurea - Lotus corniculatus, 16. Dalea candidum - Trifolium repens, 17. Desmanthus illinoensis - Medicago sativa, 18. Astragalus canadensis - Coronilla varia.*


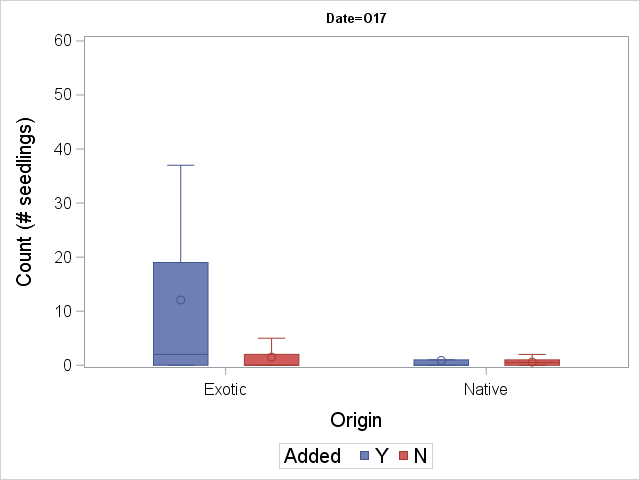

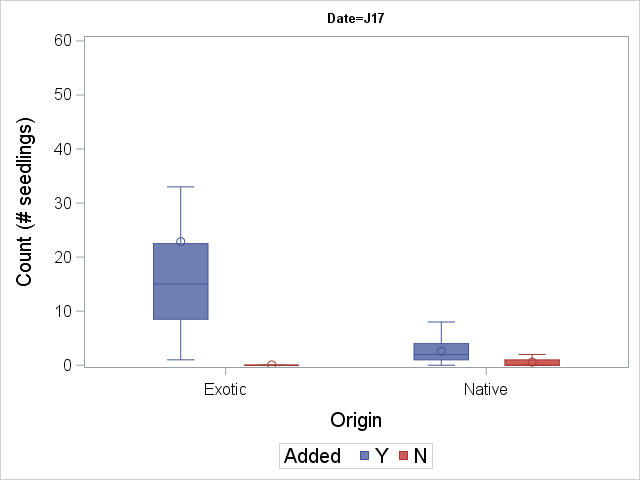


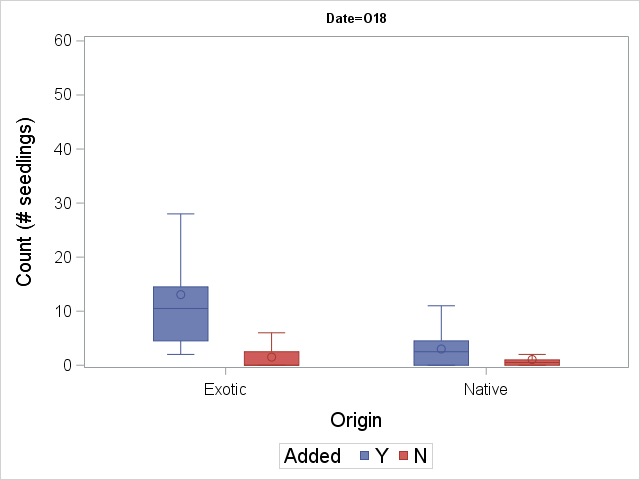

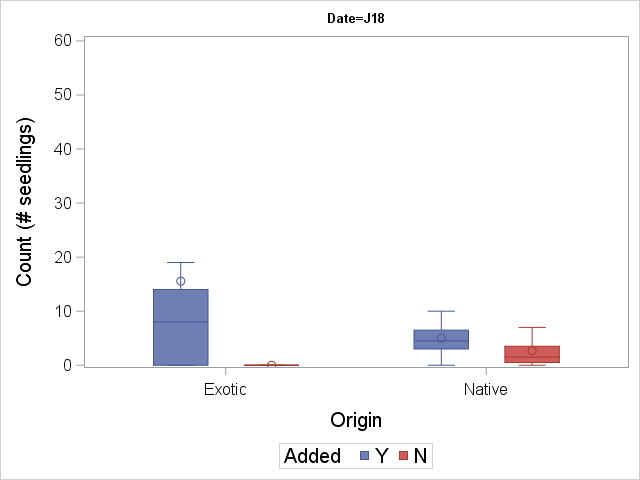


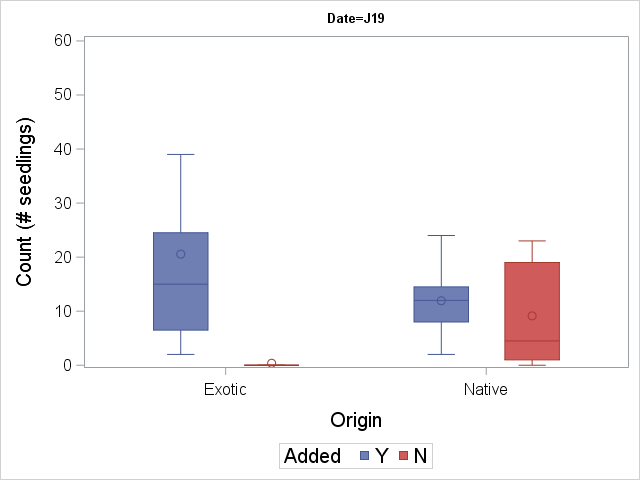


Supplemental Figure 2. Number of seedlings in mixtures for exotic species seeded into native plots (Exotic - Y) and native species seeded into exotic plots (Native - Y), compared to control plots (N).

**Table S1** List of species used in the experiment. Exotic and native species were paired based on phylogeny and growth form. Only four C_3_ grass species were used due to their paucity in the system.

| Native species | Exotic species pair | Family | | Origin | |
| --- | --- | --- | --- | --- | --- |
| *C_4_ grasses:* |  |  |  | |  |
| 1. *Schizachyrium scoparium* | *Bothriochloa ischaemum* | *Poacae* | *Asia* | |  |
| 2. *Buchloe dactyloides* | *Cynodon dactylon* | *Poacae* | *Africa* | |  |
| 3. *Sporobolus asper* | *Eragrostis curvula* | *Poacae* | *Africa* | |  |
| 4. *Panicum virgatum (short ecotype)* | *Panicum coloratum* | *Poacae* | *Africa* | |  |
| 5. *Eriochloa sericea* | *Paspalum dilatatum* | *Poacae* | *South America* | |  |
| 6. *Sorghastrum nutans* | *Sorghum halapense* | *Poacae* | *Mediterranean* | |  |
| *C_3_ grasses:* |  |  |  | |  |
| 7. *Nasella luecotricha* | *Dactylus glomerata* | *Poacae* | *Europe* | |  |
| 8. *Elymus canadensis* | *Festuca arundinacea* | *Poacae* | *Europe* | |  |
| *C_3_ Forbs:* |  |  |  | |  |
| 9. *Ratibida columnifera* | *Leucanthemum vulgare* | *Asteraceae* | *Eurasia* | |  |
| 10. *Marshallia caespitosa* | *Taraxacum officinale* | *Asteraceae* | *Europe* | |  |
| 11. *Vernonia baldwinii* | *Cichorium intybus* | *Asteraceae* | *Eurasia* | |  |
| 12. *Salvia azurea* | *Nepata cataria* | *Lamiaceae* | *Eurasia* | |  |
| 13. *Ruellia humilis* | *Ruellia brittoniana* | *Acanthaceae* | *Mexico* | |  |
| 14. *Monarda fistulosa* | *Marrubium vulgare* | *Lamiaceae* | *Eurasia* | |  |
| *C_3_ Leguminous forbs:* |  |  |  | |  |
| 15. *Dalea purpurea* | *Lotus corniculatus* | *Fabaceae* | *Eurasia* | |  |
| 16. *Dalea candidum* | *Trifolium repens* | *Fabaceae* | *Europe* | |  |
| 17. *Desmanthus illinoensis* | *Medicago sativa* | *Fabaceae* | *Asia* | |  |
| 18. *Astragalus canadensis* | *Coronilla varia* | *Fabaceae* | *Mediterranean* | |  |
